# Generic antibiotic industries: Challenges and implied strategies with regulatory perspectives

**DOI:** 10.4103/0975-7406.76481

**Published:** 2011

**Authors:** M. Venkatesh, V. G. Bairavi, K. C. Sasikumar

**Affiliations:** Regulatory Affairs, Orchid Chemicals & Pharmaceuticals Ltd., 313, Valluvar Kottam High Road, Chennai - 600 034, India

**Keywords:** Antibiotic, generic drug, Hatch-Waxman Act, US regulation

## Abstract

Ever since the discovery of antibiotics, the quality of human life greatly improved in the 20^th^ century. The discovery of penicillin transformed the medicine industry and initiated a search for a better antibiotic every time resulting in several synthetic and semi-synthetic antibiotics. Beginning with the 1937 sulfa drug tragedy, the drug regulations had a parallel growth along with the antibiotics and the antibiotic-based generic Pharma industries. This review article is focused on the scenario depicting current global Pharma industries based on generic antibiotics. Several regulatory aspects involved with these industries have been discussed along with the complexity of the market, issues that could affect their growth, their struggle for quality, and their compliance with the tightened regulations. With the skyrocketing commercialization of antibiotics through generics and the leveraging technologic renaissance, generic industries are involved in providing maximum safer benefits for the welfare of the people, highlighting its need today..

Humans are constantly exposed to a variety of microorganisms, including the major groups, namely, bacteria, fungi (yeasts and molds), algae, protozoa, and viruses. Despite steadily improving public awareness and continued diagnostic and therapeutic advances, human race faces a continuous threat from microorganisms through infections. Many individuals develop a variety of infections but quickly overcome them. In most cases these microorganisms do not produce infection because the skin and mucous membrane surfaces provide effective barriers against invasion. However, some individuals are unfortunate as a few microorganisms can invade through the barrier or through lesions from surgery or trauma and develop chronic or persistent infections. Statistics show 2.5 deaths on an average per 1 million people in the USA as a result of bacterial infections.[1] Similarly bacterial infections prevail throughout the world increasing the mortality rate considerably. Antibiotics are used to supplement the body’s natural defenses against a bacterial infection. They act either by killing bacteria or by stopping them from growing and multiplying. Antibiotics have historically played a major role in the diagnosis and treatment of infections. Antibiotics have dramatically changed the practice of medicine in this century. They have significantly reduced the occurrences of diseases, such as diphtheria, syphilis, and whooping cough.[2] A point to be noted here is that antibiotics are of high demand than any other drugs comparatively, which proves their significance in world economy as well as public health. Even though the topic is very wide through the history and can be hardly told in a nutshell, fewer literature available in this highly specified field forced an inspiration for this topic.

## History of Antibiotics

At the beginning of the 20th century, a metabolic approach was applied to the formulation of new drugs. German bacteriologist Paul Ehrlich contended that since various cells of the body and of various microorganisms could be selectively stained by certain dyes, there must be specific active groups in cells of the human body and in microorganisms to which drugs of the dye type might attach. Such a drug would then act as a “magic bullet,” attacking the target cells specifically and killing only the microorganisms, while leaving the human body unaffected.[3] Drug development made a great leap forward with the discovery of antibiotics. In 1928, the Scottish scientist Sir Alexander Fleming found a zone in a culture of bacteria that was caused by the invasion of a mold. Penicillin, the extract from that mold, was shown to cure bacterial infections. Meanwhile, in the 1930s sulfa drug became the miracle drug and was used to treat many life-threatening infections. But it tasted bad and was difficult to swallow. As a solution, a US company developed a palatable, raspberry flavored liquid product. However, they used diethylene glycol to solubilize the sulfa, which killed around 107 people, mostly children. This incident made the global countries, including the USA, the Pharma giant, to tighten their regulations. The golden age of antimicrobial therapy started in 1941 when the brilliant research of a group of investigators, led by Howard W. Florey and Ernst Chain, purified penicillin and produced quantities sufficient to permit clinical trials.[4] Subsequently, many other antibiotics have been developed. Antibiotics have almost entirely replaced sulfonamides in the treatment of bacterial infection.

## Generic Pharma Industries / Generic Medicines

The pharmaceutical industry develops and produces a variety of medicinal products that save the lives of millions of people from various diseases and permits many people suffering from illness to recover and to lead productive lives. There are different types of pharmaceutical companies, namely, mainline pharmaceutical companies, which are established firms that have many approved drugs already on the market. These companies often have a significant number of research and development (R&D) laboratories and manufacturing plants throughout the nation and around the world. In contrast, smaller pharmaceutical companies often do not have any approved drugs on the market. In addition to developing their own drugs, some of them may perform contract research for other pharmaceutical companies. Finally, generic pharmaceutical companies manufacture drugs that are no longer protected by patents. Because their products are all established drugs, they devote fewer resources to R&D and more to manufacturing.

A generic medicine is a faithful copy of a mature drug—no longer under patent—marketed with the chemical name of the active ingredient. It is a pharmaceutical product intended to be bioequivalent with the innovator or the company which launched the new drug, manufactured without a license from the innovating company and marketed after expiry of a patent or other exclusivity rights. The development of generics markets stems from at least 2 major points. On the demand side, to face the rapid growth of health care expenditures, health care systems have been reformed recently. On the supply side, however, in this decade the slow input of innovative drugs and the expiry of patents on many important medical specialties have led to increasing price competition, favoring generics in many developed countries.[5]

## Generic Antibiotics in Cost Reduction

The generic industries play a major role in the cost reduction of pharmaceutical drugs in general and antibiotics in particular. Historically, the pharmaceutical industry capitalized on the discovery that many microbial secondary metabolites act as antibiotics. Even today, significant portion of the worldwide pharmaceuticals are dedicated to the production of antibiotics. Prescription drugs represent a significant component of increasing costs, with shares ranging from 4% in the USA to nearly 18% in France and Italy.[6] The industry data shows that the average brand name prescription price in 2008 was almost 4 times the average generic price ($137.90 vs $35.22).[7] The Food and Drug Administration (FDA) analysis of 1999–2004 data shows that generic competition is associated with lower drug prices: on average, the first generic competitor prices its product only slightly lower than the brand name manufacturer; the second generic manufacturer reduces the average generic price to nearly half the brand name price; prices continue to fall but more slowly as additional generic manufacturers market the product. For products with a large number of generics, the average generic price falls to 20% of the branded price and lower.[8,9] Innovation in the pharmaceutical industry, spurred in part by competitive market forces, continues to bring enormous benefits to the world. Literature from the USA has shown that brand name manufacturers do not compete on price once generic competitors become available.[10] Because generic drugs are typically far less expensive than their corresponding brand name versions, competition from generic drugs can deliver large savings to consumers.

## United States of America – Golden Duck for Generic Antibiotics

The USA market is considered to be one of the biggest markets in the health care sector due to its million dollar business potential and is the primary target for most of the generic pharmaceutical manufacturers. In USA, the pharmaceutical industries have achieved worldwide reputation through R&D on new drugs and spend a relatively high amount of its profits on R&D compared with other industries. Each year, pharmaceutical industry testing involves millions of compounds, yet in the long run, yields only fewer new fruitful medicines. It is critical to maintain appropriate incentives for the development of new drug products, because the necessary R&D is risky and costly. At the same time, expenditures on pharmaceutical products continue to grow and often outpace expenditures for other consumer products. The new drug development, as well as the generic drug availability is well balanced by the current regulations in the USA through implementing the Hatch–Waxman Act.

In the USA, oral and parenteral antibiotics were found to possess equal shares of sales. The estimates of antibiotic availability from numerous countries throughout the world serve as an indicator of potential patterns of human antibiotics sales. The extraordinary therapeutic effects of antibiotics, the occurrence of resistance, and the considerable resources spent on antibiotics worldwide were the compelling reasons for concern about adequate and appropriate use of these powerful agents. Antibiotics often accounted for 15%–30% of drug expenditures, the largest share of expenditure among any therapeutic group of drugs. According to the US Centers for Disease Control and Prevention, 1.7 million people per year in the USA face hospital-acquired infections, leading to 5.8% deaths among them.[11] Recent figures from the USA indicate that the cost associated with the treatment of these infections in the USA is around $6.7 billion.[12] Such kind of reports infer that hospital-acquired infections in the developed world cost more than $32.5 billion, higher than the current global sales of antibiotics. Thus antibiotics having the potential impact on preventing mortality in the developed part of the world play a major impact in the global market.[12] The costs associated with these infections therefore indirectly provide an important measure of the failure of current antibiotics due to developing resistance, thus encouraging industry to search for newer antibiotics as well.

## Promotion of Generics: Hatch–Waxman Act

The FDA or USFDA is an agency of the US Department of Health and Human Services, responsible for protecting and promoting public health through the regulation and supervision of prescription and over-the-counter pharmaceutical drugs, vaccines and biopharmaceuticals, and many other commodities, which may influence public health. FDA plays an inevitable role in the approval of generic as well as new drugs, which are to be marketed in the USA. The Drug Price Competition and Patent Term Restoration Act of 1984, usually referred to as the Hatch–Waxman Act, was designed to promote generics in the USA while leaving intact a financial incentive for R&D. It allows generics to win FDA marketing approval by submitting bioequivalence studies. Approvals were generally provided with the following certifications:

- Paragraph I Certification: The generic applicant certifies that there are no patents listed in the orange book. “Orange book” being a publication of USFDA, lists the patents relating to drugs approved for marketing and sale in the USA, including patents that protect active ingredients.
- Paragraph II Certification: In case any listed patents have previously expired, the applicant may enter the marketplace immediately upon FDA approval.
- Paragraph III Certification: The applicant certifies that any listed patent has not yet expired but will expire on a particular date. The FDA may approve the Abbreviated New Drug Application (ANDA) and make it effective as of the patent expiration date.
- Paragraph IV Certification: The applicant for generic approval intends to market the drug prior to expiration of any patent(s) listed in the orange book; the applicant makes a certification that the patent(s) are not infringed or are invalid and FDA notifies the New Drug Application (NDA) holder and patent owner accordingly.[13] It also grants a period of additional marketing exclusivity to make up for the time a patented pipeline drug remains in development. This extension cannot exceed 5 years, and it is in addition to the 20 years exclusivity granted by the issuance of a patent.

Another provision of the Hatch–Waxman Act is that it grants a 30-month stay to drug companies that file suits against generic manufacturers who challene their patents. Thus the act maintains a fair balance between the innovator of a new drug and the generic drug producers.

## History of Generic Antibiotic Regulations in USA

The history of antibiotic regulation clarifies the relationship between regulatory plan and the scientific/regulatory constraints and the marketing conditions in which they operate. Antibiotics and insulin-containing drugs were added to the regulatory scheme beginning with a series of steps in 1941. However, the procedures for establishing safety and efficacy applicable to other “new drug” antibiotics were subject to a far different regulatory scheme.

In the case of antibiotics, the monographs were developed on the basis of the first product or the innovator product reviewed and approved in the antibiotic class. Thereafter, any forthcoming vendor merely needed to show that it was bioequivalent to the innovator product for which the monograph was developed and that it followed the specifications of the monograph. In this way, the innovator owned no rights even though the following vendors receive the approval based on his certificate. This seems to be a paradox.[14]

Along with this, the antibiotic vendor should provide a sample of each batch of the antibiotics to the FDA for laboratory testing and certification. The batches were tested by the agency and if found to meet the standards, the Antibiotic Certificate was issued.[15] Also, the batches which were not tested but released prior to such certification were considered as misbranded. Quite unexpected by the agency, the testing of antibiotics became slower due to practical difficulties, such as personnel and facilities limitations. As a result, large quantities of antibiotic products were held in quarantine for many weeks just only for the clearance by the agencies. As an initiative to relax the regulations, in 1980, the FDA announced that testing of topical antibiotics would no longer be required. Finally, in 1982, the batch certification program for antibiotics was eliminated entirely but was considered and regulated as for any other drug to comply with the monograph.[16]

In 1986, over-the-counter antibiotics that complied with the applicable monograph were excluded from the batch certification process. In contrast to the earlier times where only penicillin was the available antibiotic in the market, several hundreds of antibiotics started getting approval from the agency. As a result of the 1962 Amendments, the FDA required the submission for several antibiotics of scientific evidence of substantial well-controlled clinical studies, demonstrating the effectiveness of the product. Those products that failed to provide such evidence had their certifications overturned. In addition, the FDA cancelled approval of several antibiotics that did not have substantial scientific evidence. In 1985, antibiotic applications were classed as either “New Antibiotic Drug Applications” or “Abbreviated Antibiotic Drug Applications.” In line with the new regulatory framework, applicants and manufacturers could now make certain changes in their products without requiring prior FDA approval.

In 1997, antibiotics were started to be reviewed and approved on the same basis as any other pharmaceutical product. In 2000, the FDA recounted the term “antibiotic drug” as it refers not only to the active chemical substance, but to any derivative of the substance, such as a salt or an ester of the substance. Under the new scheme, similar to nonantibiotics, antibiotics could also henceforth contain drug substance information in the finished product application itself. Thus, antibiotics entered into the normal stream of pharmaceuticals as any other pharmaceutical categories.

## Global Scenario of Antibiotics

The antibiotics market generated sales of US$42 billion in 2009 globally, with 14 products recording sales of more than $1 billion.[17,18] There were 7 blockbuster antibacterial drugs and 8 antivirals. The volume of antibiotic use was increasing in most European countries between 1997 and 2010. In European countries, penicillins were the most prescribed outpatient antibiotics and further enlarged their leading position between 1997 and 2003.[19] Likewise, the use of quinolones surged, while the use of another 2 major classes of antibiotics, tetracyclines and sulfonamides, stagnated or decreased in most European countries as newer antibiotics superseded them. More detailed data on the European antibiotic markets are not available due to the wide differences in consumption. Many reasons have been proposed to explain the large differences in the consumption of antibacterial agents among European countries, including the incidence of community-acquired infections, knowledge about antibiotics, and regulatory practices. Striking geographic variations were observed in the use of various antibiotic classes. For instance, the narrowspectrum penicillins and the first-generation cephalosporins were widely prescribed for the treatment of community-acquired infections in many Nordic countries, while they almost disappeared in most Southern European countries. In the latter, an increased use of the newer antibiotics, such as amoxicillin/clavulanic acid, macrolides, and quinolones was also observed.

Japan is the second-largest pharmaceutical market in the world. It is well known that Japan is the third country next to the USA and the UK to become self-sufficient in penicillin manufacture as early as in 1948. Besides this, much effort was made in exploratory research on anti-infectives. Starting from colistin, several antibiotics, namely, kanamycin, bleomycin, piperacillin, norfloxacin, meropenem, and others, are from Japan. As of the 12 months to Q1 2009, the Japanese pharmaceutical market accounted for nearly 10% of the world market, and generated total sales of $71.6 billion. The Japanese government ensures that all citizens and workers have health insurance. Compared with that in 2008, Japan tightened monitoring and inspection of imported antibiotics in 2009 with more stringent requirements for their testing.

## Regulatory Challenges

**Figure 1:**
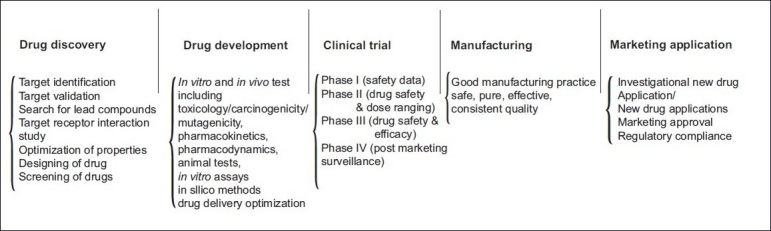
Antibiotics from generic industry to regulated market

Several regulatory challenges were involved in the development of a generic antibiotic. Regulatory challenges, such as bioequivalence, patent expiry, newer antibiotic, and the complexity involved in the regulated market, are explained in the following sections. Figure 1 depicts the various tasks involved with the drug development.

### Bioequivalence

In order to protect consumers, generic products must be demonstrated to be therapeutically equivalent to a previously approved product, typically an innovator product. Nowadays bioequivalence studies are essential part of a company’s registration dossier. These bioequivalence studies measure the bioavailability of 2 formulations of the same active ingredient. The plasma concentration of a drug determines the number of drug molecules at a receptor and hence the therapeutic effect. The plasma concentration is governed by absorption, distribution, metabolism, and elimination. The pharmacokinetic parameters measured are area-under-the-concentration (AUC) time curve, the peak concentration (C_max_), and the time to peak concentration (t_max_). Statistically, geometric mean ratio of the test to the reference drug for AUC and C_max_ must fall within 90% confidence limits of 80 and 125.[20,21] Within these statistical limits, these particular parameters will be sufficient for bioequivalence.

Substitution of generic drugs for brand name products is highly controversial, especially in antibiotics and often is met with suspicion by health care providers and patients. Bioequivalence issues present more concerns over generic drug substitution, such as consumer perception of risk, differences in product and packaging appearance, and differences in excipients. Also, several antibiotics, such as cephalosporins, have relatively narrow therapeutic window and would undergo stringent bioequivalence testing, because relatively modest changes in the concentration achieved in body fluids might well be associated with large changes in the frequency of therapeutic failure or significant toxicity. It arises several questions as whether is it possible to market various intravenous products with same concentration of active ingredient but with variable quality, even if it does not affect the clinical outcome, it does little to build trust in the generic industry.[22]

### Patent expiry

As the earth moves closer to the concept of a global village by elimination of trade barriers, new challenges crop up. A case in point is the market for generic antibiotics. Hatch–Waxman established a regulatory framework that sought to balance incentives for continued innovation by brand name companies and opportunities for market entry by generic drugs. Before 1962, it was observed in the USA that out of the 150 off patent drugs in the market, there were no generic drugs. Many companies did not go in for manufacture of generic drugs because of the impractical and nonscientific manner in which the regulatory authorities viewed the approval process. The Hatch–Waxman Act addressed these issues and proposed many reforms. The underlying objective of the Act is that in the absence of generic drugs, it is difficult to check the profiteering motive of the patent owner of a drug, who may put patients to ransom. However, at the same time it also takes care of the interest of the patent owner and provides relief for undue lengthy process. It is now possible for many generic companies to qualify for the 180-day market exclusivity if several applications are filed on the same day. Under the Hatch–Waxman Act, the government has a system of patent term “restorations” under which monopoly of the original patentee can be extended for a maximum period of 5 years in addition to the initial patent term. In the European Union also there exists a system of supplementary protection.[23]

### Newer antibiotics and their hurdles

Although the need for new antibiotics is increasing, a number of factors make these drugs less economically attractive than drugs that treat chronic diseases. Pharmaceutical companies appear to be less interested in developing anti-infective drugs.[24] Reason being antibiotics are typically taken for a week or 2, which makes them cost-effective for the health system, but less lucrative to drug companies than medicines for diseases, such as cancer or diabetes, which might be taken for months together.[25] In addition, to prevent the evolution of resistant strains of bacteria, physicians who treat infectious diseases try very hard to limit the overuse of newer antibiotics. These being the reason for the struggle of existence for the newer antibiotics, the discovery of newer antibiotics came to a slower pace due to loss of interest. Such a condition makes a patient suffer from antibiotic resistance and costly medication. Literature search shows that the FDA approvals of new antibiotics declined 56% during the past 20 years (1998-2002 vs 1983-1987).[26,27] Further, to prove the condition, only a few new antibiotics were in the pipeline or already approved by the FDA. Table 1shows the new drug applications approved from 2005 to 2009.

**Table 1:** New drug applications (antibiotics) approved during the period 2005–2009

Antibiotics new drug approvals during 2005–2009^[30]^					
NDA number	Proprietary name	Established name	Applicant	Approval date	Indication
N022106	Doribax	Doripenem	Ortho McNeil Janssen	Oct 12, 2007	Provides for the treatment of complicated intraabdominal and complicated urinary tract infections caused by susceptible isolates of the designated microorganisms
N021821	Tygacil	Tigecycline	Wyeth Pharms Ltd	Jun 15, 2005	Tygacil is indicated for the treatment of complicated skin and skin structure Infections and complicated intraabdominal infections
N050786	Pylera	Biskalcitrate; metronidazole; tetracycline hydrochloride	Axcan candipharm	Sep 28, 2006	Provides for the treatment of patients with *Helicobacter pylori* infection and duodenal ulcer disease (active or history of within the past 5 years) to eradicate *H. pylori*
N050818	Tobradex St	Tobramycin/dexamethasone	Alcon	Feb 13, 2009	Superficial bacterial ocular infection or a risk of bacterial ocular infection exists

### Complexity of the market

**Figure 2:**
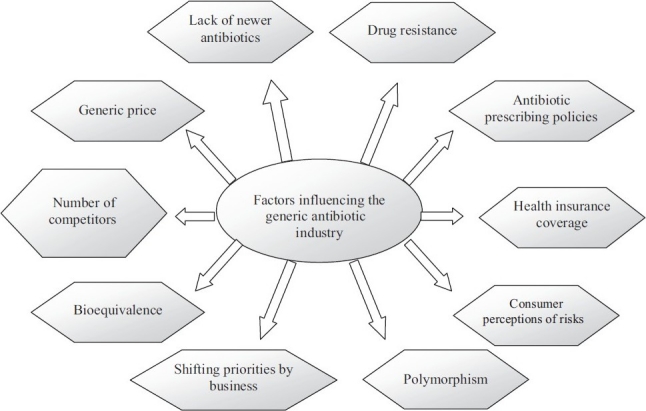
Factors influencing the generic antibiotic market growth

Even though it looks simpler, the markets for generic antibiotics are more complex due to several factors. Several factors that influence the generic antibiotic market growth are depicted in Figure 2. Long-term growth in the global antibiotics market would be affected by 2 major factors: antibiotic resistance and generic competition.

The evolution of antibacterial resistance in human pathogenic microorganisms is the result of the interaction between antibiotic exposure and the transmission of resistance within and between individuals. In the community, there is cumulative evidence that the antibiotic exposure of populations promotes acquired antimicrobial resistance in community pathogens, such as *Streptococcus pyogenes*, cutaneous staphylococci, and propionibacteria. In a hospital setting, an increased use of antibiotics is often associated with an increase in the frequency of antibiotic resistance. Furthermore, the relationship between antibiotic use and resistance is most evident when resistance is due to mutations selected during therapy, resulting in clinical failure. The inappropriate use or overuse of antibiotics has caused the major problem of polydrug resistance, and resistance patterns of bacteria to the new antibiotics are likely to parallel the extent to which they prescribe. Such general antibiotic resistance affects the market to a greater extent.[28] In today’s world, even if the innovator obtains regulatory approval to market a newer antibiotic, its commercial success will be limited, as the regulatory agencies are likely to move forward on a recommendation for the sale of cheaper generic copies of the same drug to the public. Furthermore, in reality, newer antibiotics are kept in reserve so as to avoid resistance from the microorganisms. So a physician will not wait around deciding whether to prescribe the newer antibiotic or the older generic. They are likely to prescribe a generic anyway, in the hopes that the patient is among the two-thirds of the population who will get a greater protection against relapse.

In the USA, a black box warning is a type of warning that appears on the package insert for prescription drugs that may cause serious adverse effects. It is so named for the black border that usually surrounds the text of the warning. Several antibiotics, including ciprofloxacin, gemifloxacin, levofloxacin, moxifloxacin, norfloxacin, and ofloxacin are affected by these blackbox warnings. Similarly “Dear Doctor” letter is intended to alert physicians to safety precautions that should be taken to reduce the potential risk reported to be associated with the drug products. Even though these warnings may be common for both the generic and branded drugs, these label warnings may create a negative impact on the public perception with the generic antibiotics. Apart from these, drug withdrawal and recall procedures affect the generic market in varying degrees. Recalls are an appropriate alternative method for removing or correcting marketed consumer products, their labeling, and/or promotional literature that violate the laws administered by the FDA.[29] Recalls may be conducted on a firm’s own initiative, by FDA request or by FDA order, under statutory authority. Such type of FDA’s activities affect the generic market mainly due to media coverage and are considered to be serious issues.

Pharmaceutical firms that produce an antibiotic are usually given temporary monopoly power through a patent, granted to recover the incurred investment in R&D and by this to encourage future innovation of new drugs. The granting of this monopoly power ignores the fact that this also gives them some control over the levels of the drug’s treatment efficacy on the one hand, as well as of the infected population on the other. In turn, a too intensive use of antibiotics within the community may lead to an increase in the bacterial resistance of the drug. Furthermore, literature from the USA has shown that brand name manufacturers do not compete on price once generic competitors become available. The lack of price competition may lead to increased costs in the private market. Private insurance companies generally do not require generic substitution and some provinces do not require generic substitution for cash-paying customers.[17] Furthermore, increasingly complex clinical trials are now required to gain approval, with different requirements in the USA and the European Union. These hurdles and drawbacks make the market a more complex one to predict, but still a setback lays for the discovery of newer antibiotics.

## Discussion and Conclusion

Antibiotics are among the most frequently prescribed medications in modern medicine. Most antibiotics have 2 names, the trade or brand name, created by the drug company that manufactures the drug or otherwise known as innovator, and a generic name, based on the antibiotic’s chemical structure or chemical class. The antibiotic market has evolved itself to a much stable place in spite of its complexity. The regulations of global health communities or agencies have been strictly developed on these antibiotics due to their potential in curing life-threatening diseases. Previously, a special regulatory care was given to antibiotics rather than any other drugs due to their relatively newness and also due to their significance in usage by public for life-saving purpose. Antibiotics were subjected to a batch certification requirement apart from complying with the monograph. This regulation required that each batch of antibiotic produced be certified to conform to the regulations of identity, strength, quality, and purity before marketing in the USA. This resulted in an aggressive cost increase in the antibiotic sector as well as time delay for approval. Nowadays, after several amendments to the regulations by the FDA, antibiotics are being regulated as any other pharmaceuticals. At the same time, industries are not showing interest in discovering newer antibiotics due to several factors, such as drug resistance, cost involved in the research, comparatively less profit due to industrial competencies, and so on. The introduction of Hatch–Waxman Act has made a paradigm shift in the industry by balancing the benefits for both the innovator and the generic industry. The introduction of only few newer antibiotics in the last 5 years shows the lack of awareness about these antibiotics. Although the infections were inhibited when improved sanitation and drugs were applied to combat microbes, and while many of the terrifying acute diseases, such as typhoid, cholera, and dysentery, have been subdued, many serious microbial diseases have not been eliminated. With the dangerously growing levels of antibiotic resistance and newer type of infections, organisms, and so on, it emphasizes the need for a more streamlined and enhanced means of developing and approving new agents, the need for greater integration of oversight and policy development efforts, and the necessity of greater availability of better data. Rather, regulations should be tightened up to avoid improper prescriptions and the misuse of these antibiotics.

However, the introduction of Hatch–Waxman Act has in fact increased the generic antibiotic industries and has a definite effect on their growth. The law requires that these drugs must meet the specifications of the official compendia, as any other pharmaceuticals. Although several efforts have been taken by the regulatory agencies devoted to the tests and methods of assay of antibiotics, including sterility, biological test, microbiological and chemical assays, general and specific chemical tests, and tests on specific dosage forms, the generic industries struggle hard to bring the quality and safe antibiotics complying with these regulations. In future, in spite of the complexity and competency in the field, the wellbeing of the generic as well as the industries involved with the new drug inventions should be well protected to preserve the public health.

In this article, generic Pharma industries with respect to antibiotics were explored. Considering the Hatch–Waxman Act, how the generic approvals made as fair has been explained. Also, history of antibiotics, its regulations, current market scenario, and several regulatory challenges facing antibiotic industries have also been discussed with an effort to bring into light a specialized field with challenges. However, with the increase in globalization and the demand for newer antibiotics, industries should be encouraged with focus on the future regarding the antibiotic. In summary, it is critical to devise strategies for antibiotic regulations, to ensure public health with wellbalanced commercial and regulatory aspects.[30]
